# Hidden Cost of Poor Quality in Laboratory Medicine: From Errors and Waste to Value-Based Care

**DOI:** 10.7759/cureus.117933

**Published:** 2026-10-14

**Authors:** Elsa Faviou, Christina Economidou, Ileana V Baltogianni, Nikolaos Baltogiannis

**Affiliations:** 1 Biopathology and Laboratory Medicine, Affidea Diagnostic Center, Athens, GRC; 2 Anesthesiology, University of Wisconsin School of Medicine and Public Health, Madison, USA; 3 Pediatric Surgery, Pediatrics Center of Athens, Athens Medical Group, Athens, GRC

**Keywords:** cost of quality, laboratory errors, laboratory medicine, quality indicators, test utilization, value-based laboratory medicine

## Abstract

To examine where poor laboratory quality generates economic and clinical consequences across the total testing process and to propose a practical framework for identifying preventable waste.

This narrative review draws on the focused PubMed/MEDLINE search described in the original submission, reference-list checking, and a separately documented PubMed update performed on September 29, 2026. Eight topic-specific strategies used title/abstract terms and Medical Subject Headings; publications were selected for direct relevance to laboratory quality failure, resource use, test utilization, and value-based care. No pooled analysis or formal risk-of-bias assessment was performed.

Preanalytical defects, repeated work, and inappropriate testing consume resources across laboratory and clinical workflows. Published monetary estimates depend on study setting and costing perspective, while evidence on downstream patient outcomes remains less complete.

Our conceptual Cost of Poor Laboratory Quality (COPLQ) framework links recurrent, measurable failures to local resource use and clinical consequences. Prospective evaluation is required before it can be used as a validated economic model.

## Introduction and background

Clinical laboratories are expected to provide accurate and timely information while managing increasing workloads, technological complexity, workforce constraints, and expenditure. In 2024, US Medicare Part B payments for approximately 380 million clinical diagnostic laboratory tests under the Clinical Laboratory Fee Schedule totaled US$8.4 billion [1]. This is a single US public payer’s payments, including genetic tests, not worldwide laboratory expenditure or the cost of quality failure. The total testing process spans test selection and ordering, specimen collection and transport, analytical measurement, result validation and reporting, and the clinical interpretation and use of the result. Efficiency is often discussed in terms of reagent prices, cost per test, staffing, or throughput; these measures capture only part of the resources used.

Poor quality also consumes resources. A rejected specimen requires recollection and reprocessing. A failed analytical run consumes reagents and technical time. Correcting a report requires investigation and communication. A delayed result may leave diagnostic or therapeutic decisions pending. Once a failure enters the clinical pathway, its consequences may include repeat investigations, inappropriate or delayed treatment, and additional use of healthcare resources. The economic consequences of poor specimen quality have been modeled at laboratory, institutional, and patient-care levels [2].

This distinction is central to the Cost of Quality (CoQ) approach, which separates expenditure on prevention and appraisal from costs generated by internal and external failures. In laboratory and pathology specimen processes, the framework can make prevention, appraisal, and failure costs visible [3]. Quality-control materials, maintenance, and training appear clearly in budgets, whereas repeated work, recollection, and investigation can be distributed across services.

At the same time, the concept of laboratory value has evolved. Value-based laboratory medicine shifts attention from activity and unit cost toward the contribution of laboratory information to clinical decisions, patient outcomes, safety, and appropriate resource use [4,5]. A recent value-based score for clinical laboratories further integrates quality indicators, traceability, automation, information management, and interaction with clinicians [6]. A subsequent pilot in 12 European laboratories found variation in value-based scores, with clinical interaction consistently the weakest domain [7]. The invited sites do not establish representative European benchmarks.

The economic discussion therefore needs to move beyond the price of an individual test. A technically correct result may still represent poor value if the test was unnecessary, duplicated, performed at an inappropriate interval, or reported too late to influence care. Conversely, expenditure on prevention may represent good value when it avoids recurrent failure.

This review traces the economic consequences of failure across the testing process and examines how quality indicators and targeted interventions can make recurrent loss visible. It proposes a practical Cost of Poor Laboratory Quality (COPLQ) framework for local assessment of resources consumed by poor quality.

## Review

Narrative review approach

The original submission described a focused narrative PubMed/MEDLINE search emphasizing publications from January 2015 through August 2026, with earlier foundational articles and relevant references retained. Its reported concepts included cost of quality, laboratory and preanalytical errors, quality indicators, inappropriate testing, utilization, automation, autoverification, artificial intelligence (AI), and value-based laboratory medicine. Exact original query strings, execution dates, and counts were not retained in the materials available for this revision; the revision search below is reported separately and is not attributed retrospectively to the original search. During revision, eight topic-specific PubMed searches were executed on September 29, 2026, combining title/abstract terms ([tiab]) and Medical Subject Headings ([mh]) with Boolean operators. The cost-of-quality strategy had no publication-date limit; the other seven covered January 1, 2015, through September 29, 2026 ([dp]). All eight were limited to English ([la]). The final union retrieved 2,190 unique PubMed records. The entered queries, including the PubMed History reference used for the refined automation search, and retrieval counts are provided in Appendices A-B.

Publications were considered for direct relevance to the economic, operational, or clinical consequences of laboratory quality failure. Priority was given to original studies with defined settings and outcomes, professional recommendations, and reviews directly informing the framework. Five research articles newly cited during revision (the European pilot, Eker, Panteghini, Eaton, and Corchon-Peyrallo studies) were present in the 2,190-record PubMed export; the Lean Six Sigma study was identified by targeted citation searching. The government expenditure report was identified separately [1]. During revision, the manuscript was checked against the six SANRA items: justification of the review’s importance, statement of its aims, description of the literature search, referencing, scientific reasoning, and presentation of relevant endpoint data [8]. No formal SANRA score was assigned. Study design, costing perspective, outcome definition, and setting informed the descriptive synthesis. This was not an exhaustive systematic review: no formal risk-of-bias tool, pooled analysis, or PRISMA study-selection process was applied. The 2,190 retrievals must not be interpreted as records individually screened or studies included.

The evidence was organized around four questions: where failure occurs, what resources it consumes, how it can be measured, and which interventions may reduce recurrence. Monetary results are attributed to individual studies rather than pooled across different prices, workflows, and perspectives. Table 1 distinguishes the original approach from the revised update. The complete revision search is given in Appendices A-B.

**Table 1:** Literature search strategy and study selection approach. This focused narrative review did not use a PRISMA-based study-selection process, perform a pooled analysis, or conduct a formal risk-of-bias assessment. The 2,190 records represent the total number of unique records retrieved, rather than the number of records screened or studies included. PRISMA, Preferred Reporting Items for Systematic Reviews and Meta-Analyses

Source	Search period	Search concepts/terms	Selection approach
Original focused PubMed/MEDLINE search, as described in the initial submission	January 2015-August 2026 emphasis; earlier foundational work	Reported search concepts; exact original strings, execution date, and counts unavailable in retained materials	Direct relevance and priority for original evidence, recommendations, and pertinent reviews
Reference-list screening and targeted citation searching	No separate date restriction	Reference lists of, and citations to, key articles identified during the PubMed/MEDLINE search	Additional relevant papers, including the Lean Six Sigma study, identified by citation searching
Revision PubMed search	Executed September 29, 2026; S1 unrestricted date; S2-S8 January 2015-September 2026; English	Eight complete Boolean expressions with [tiab], [mh], [dp], [la] in Appendices A-B; 2,190 unique records in the final union	Targeted narrative selection by topic, design, outcomes, costing perspective, and applicability
Government expenditure report	US Medicare Part B, 2024 claims	OIG report OEI-09-25-00330, January 2026	Single-payer expenditure context; not global spending or cost of poor quality

From cost of quality to laboratory value

The CoQ model separates expenditure into prevention, appraisal, internal failure, and external failure. Applied to laboratory medicine, this distinction is useful because it separates resources deliberately invested in a reliable process from those consumed because the process did not perform as intended [3].

Prevention includes staff training, competency assessment, method verification, process standardization, preventive maintenance, and appropriate specimen-collection procedures. Appraisal includes internal quality control, external quality assessment, audits, and performance monitoring. These activities are visible and are therefore easy to question when budgets are constrained.

Internal failure is less visible. It includes rejected specimens, failed analytical runs, unnecessary repeat testing, wasted reagents, troubleshooting, and work performed twice without adding clinical information. External failure begins when an inadequate laboratory service has already reached the user, for example through an incorrect or corrected report, delayed communication of a critical result, or a misleading result that has entered clinical decision-making. Table 2 presents the authors’ synthesis of how these four CoQ components apply to routine laboratory practice.

**Table 2:** Applying the cost of quality framework to laboratory practice. Source: Authors’ original synthesis based on the cost of quality concepts discussed in Carlson et al. [3]. No table, figure, or verbatim content has been reproduced or adapted from the original publication. QC, quality control; EQA, external quality assessment

CoQ component	Role in laboratory practice	Representative activities or failures	Economic interpretation
Prevention	Activities designed to reduce the likelihood of error before it occurs	Staff competency, standardized collection procedures, method verification, preventive maintenance	Resources are invested prospectively to reduce recurrent failure and avoid downstream rework
Appraisal	Activities used to detect deviation before an unreliable result reaches clinical care	Internal quality control, external quality assessment, audits, performance monitoring	Resources are used to identify instability early and limit the consequences of undetected error.
Internal failure	Failures identified before the laboratory service reaches the clinical user.	Rejected specimens, failed runs, repeat analysis, troubleshooting, reagent loss, downtime.	Resources are consumed in rework without generating additional clinical value.
External failure	Failures recognized after a result or service has reached the clinical pathway.	Corrected reports, delayed critical-result communication, patient recall, investigation of misleading results	Costs may extend beyond the laboratory through additional clinical work, delay, and organizational consequences.

Prevention is not inherently cheap, and more quality activity does not automatically mean better quality: poorly designed control procedures, redundant documentation, and unnecessary manual review consume resources too. What matters is whether spending targets failures that are frequent, costly, preventable, or clinically significant enough to justify the effort.

This perspective aligns naturally with value-based laboratory medicine. Contemporary definitions of value emphasize the effectiveness of laboratory information in improving outcomes while optimizing resources, rather than simply increasing throughput or reducing the cost of each result [4,5]. Lower expenditure and higher value are therefore not synonymous. Reducing preventive maintenance or quality-control activity may lower a visible budget line while increasing the probability of failure elsewhere. Conversely, an intervention that reduces recurrent specimen errors may release resources across nursing, transport, laboratory, and clinical services.

Preanalytical phase

Despite major improvements in analytical systems, the preanalytical phase remains vulnerable. In one core laboratory that retrospectively captured errors from January 2022 to May 2023, Lin et al. classified 85,894 of 87,317 recorded errors (98.4%) as preanalytical [9]. This is the distribution among errors identified by that laboratory’s methods, not a general estimate across all settings. Hemolysis was a major contributor; therefore, collection, identification, and transport remain practical priorities.

Common examples include patient-identification errors, incorrect tubes, insufficient volume, hemolysis, clotting, contamination, labeling errors, and transport delays. Recent reviews continue to emphasize that prevention requires cooperation between laboratory and clinical staff because much of the preanalytical process occurs outside direct laboratory control [10].

A rejected specimen illustrates why direct analytical cost is a poor estimate of the true burden. The discarded tube and reagents represent only part of the loss. Recollection requires another patient interaction, staff time, transport, accessioning, processing, and additional time before the result becomes available. In emergency and inpatient settings, the delay may also affect time-sensitive clinical decisions. For patients, recollection adds discomfort, inconvenience, and sometimes anxiety, consequences that are difficult to monetize but remain clinically relevant.

Hemolysis is similarly important because it is both common and analyte-dependent. Depending on the degree of interference, it may lead to suppressed or spurious results, recollection, additional communication, or further clinical clarification. Even a modest cost per episode can become substantial when an event is repeated at scale.

Evidence also shows that preanalytical improvement can be evaluated economically. Foglia et al. reported that integrated venous blood collection with full sample traceability at one Italian university hospital reduced preanalytical errors and was associated with a 12.7% cost saving per tube [11]. The monetary effect cannot be transferred directly to other laboratories, but the study demonstrates that preanalytical quality can be assessed in terms of both error reduction and resource use.

A retrospective analysis of 2019 data from one 836-bed Turkish hospital, including samples sent to a central laboratory, estimated TRY 438,284.51 (€68,918.07) in direct costs associated with 32,783 preanalytical sample errors, including materials, logistics, transfer, personnel, and waste [12]. The estimate excludes unmeasured downstream clinical costs and cannot be transferred directly to other settings.

Analytical phase

Analytical errors are less frequent than extra-analytical failures in the contemporary core laboratory study [9], but an undetected analytical problem can affect a sequence of patient results.

Internal quality control (IQC), calibration, method verification, external quality assessment, and preventive maintenance therefore have an economic role in addition to their technical role. Their purpose is to identify instability before it propagates into released patient results. Recent recommendations from the International Federation of Clinical Chemistry and Laboratory Medicine (IFCC), aligned with International Organization for Standardization (ISO) 15189:2022, support a risk-based approach to IQC, including appropriate control frequency, acceptance limits, and management of nonconformities [13].

Process design can also prevent unnecessary appraisal expenditure. In a Canadian laboratory Lean Six Sigma quality-control project, annual spending on control materials fell from CAD 346,395 to CAD 255,267 after workflow and QC-plan changes [14]. This local before-and-after result illustrates how a risk-based quality plan can reduce redundant consumption without implying a universal saving.

When an analytical problem is detected late, the resulting work can include repeat controls, recalibration, reagent loss, repeat patient analysis, instrument downtime, review of previously released results, corrected reports, and communication with clinicians. The meaningful comparison is therefore not the cost of quality control against zero expenditure, but the cost of an appropriate control strategy against the probability and consequences of undetected analytical failure.

Postanalytical phase

Postanalytical quality may be underestimated because the measurement itself has already been completed. Clinically, however, an accurate result has value only if it is reported correctly, reaches the appropriate recipient, and is available in time to influence management.

Delayed turnaround time (TAT), transcription errors, incorrect reports, delayed communication of critical values, and inappropriate validation are therefore genuine quality failures. Their direct laboratory costs may be small, whereas their downstream effects may be larger, including repeated calls, delayed decisions, prolonged patient pathways, or empirical management while information is pending.

Contemporary quality frameworks accordingly extend beyond analytical imprecision. The IFCC Model of Quality Indicators was developed to monitor critical steps across the total testing process [15], and the more recent Essential Quality Indicators Panel includes sample/request identification, specimen rejection, hemolysis, external quality assessment performance, emergency troponin TAT, and incorrect reports [16].

Hidden cost of failure

The main difficulty in estimating the cost of poor quality is fragmentation. Laboratories usually know what they spend on reagents, controls, maintenance, and staffing. They rarely know how much time is spent each year on rejected samples, repeat testing, specimen tracing, investigation of discrepant results, corrected reports, or avoidable calls from clinical units.

For practical purposes, the cost of a quality failure can be considered at four levels. The first is direct laboratory cost: tubes, reagents, consumables, controls, and analyzer time. The second is workforce and workflow cost: laboratory staff, phlebotomy or nursing time, transport, investigation, supervision, and communication. The third is downstream clinical cost: additional testing, reassessment, delayed treatment, or prolonged care. The fourth includes consequences that are difficult to monetize, such as patient inconvenience, loss of confidence, reputational damage, and medicolegal exposure.

A laboratory does not need a sophisticated health-economic model to begin examining these losses. A workable starting point is the annual number of recurrent events multiplied by the locally estimated resources consumed per event. The event should be defined broadly. For a rejected specimen, the relevant cost should not stop at the tube; for a corrected report, investigation and communication may matter more than reagent expenditure.

The same logic applies to recurrent minor failures. Rare severe events deserve attention because of patient risk, but small defects repeated thousands of times may represent a substantial cumulative burden. Prioritization should therefore consider frequency, preventability, resource use, and clinical risk rather than frequency alone. Table 3 links common laboratory failures with measurable indicators, hidden resource use, clinical consequences, and possible interventions.

**Table 3:** Linking quality failures to measurable indicators and resource use. Source: Authors’ illustrative synthesis informed by the studies and recommendations discussed in the text. Costs and interventions are setting-specific and should be adapted locally. HIL, hemolysis/icterus/lipemia; IQC, internal quality control; TAT, turnaround time

Quality failure	Measurable indicator	Main hidden cost	Potential clinical consequence	Targeted intervention
Hemolysis	Hemolysis rate	Recollection, processing, staff time	Delayed or misleading result	Collection training; HIL monitoring
Insufficient/wrong specimen	Rejection rate by cause	Repeat collection, transport, accessioning	Diagnostic delay	Standardized collection requirements
Patient/sample misidentification	Misidentification rate	Investigation and recollection	Potential serious diagnostic error	Positive identification; barcode systems
Failed analytical run	Rejected run/QC failure rate	Reagents, downtime, repeat testing	Delayed reporting	Risk-based IQC; maintenance
Unnecessary repeat analysis	Repeat-analysis rate	Reagents and staff time	Increased TAT	Validated repeat rules; autoverification
Corrected report	Corrected-report rate	Investigation, documentation, communication	Clinical reassessment	Validation rules; process review
Prolonged TAT	Percentage outside target	Workflow disruption, calls	Delayed decision-making	Workflow redesign; automation
Delayed critical result	Critical-result notification time	Escalation and communication	Delayed urgent treatment	Closed-loop notification
Inappropriate repeat ordering	Tests outside retesting interval	Test cost and downstream work-up	Low-value care	Demand-management rules

Inappropriate testing: poor quality without analytical errors

Laboratory quality cannot be restricted to whether a measurement is analytically correct. A technically flawless result may still have little value if the test was unnecessary, duplicated, performed too soon, or unlikely to change management.

The scale of this problem can be substantial. In an analysis of 2019 US outpatient claims, Smart et al. found that 7% to 51% of tests exceeded recommended frequencies, depending on the test; for four common tests (25-hydroxyvitamin D, prostate-specific antigen (PSA), lipid panel, and glycated hemoglobin (HbA1c)), their conservative estimate of direct excess expenditure exceeded US$350 million [17]. Claims-based estimates do not capture all clinical context and cannot be transferred directly to other systems.

Demand-management strategies include electronic order-entry rules, reflex testing, and minimum retesting intervals. Panteghini et al. described 15 years of laboratory-led appropriateness interventions, including computerized physician order entry (CPOE)-based measures, at one academic institution [18], while Eaton et al. synthesized Choosing Wisely-informed approaches to repetitive inpatient testing [19]. In a prospective longitudinal before-and-after study from one Valencia hospital laboratory serving 154 primary care physicians across 28 centers, a laboratory-led CPOE system was associated with a €5.69 (14%) lower median cost per request and a median reduction of 2.2 tests per request [20]. The before-and-after design does not, by itself, establish that the intervention caused all observed changes.

The cost of inappropriate testing is not limited to the test itself. Borderline or incidental abnormalities may lead to further measurements, specialist review, or imaging. Repeated testing also creates workload in both laboratory and clinical services without necessarily adding information.

Demand management should not, however, be reduced to ordering fewer tests. Underutilization is also poor value when a clinically indicated test is omitted or delayed. The objective is appropriate use: the right investigation for the right patient at a clinically useful time. Laboratory professionals can contribute through minimum retesting intervals, reflex and reflective testing, electronic decision support, review of low-value tests, and direct consultation with clinical teams. In this role, the laboratory becomes part of diagnostic stewardship rather than a passive producer of results.

Making poor quality measurable

Quality indicators provide the most practical starting point for economic assessment because many relevant events are already counted. Sciacovelli et al. have shown their value in monitoring vulnerabilities across the total testing process and benchmarking areas that conventional analytical quality control does not capture [21]. The recent IFCC Essential Quality Indicators Panel provides a focused set of measures directly relevant to patient safety [16].

Once an event is measured, the laboratory can examine the resources consumed each time it occurs. A rejection rate can be linked to recollection and reprocessing; a corrected-report rate to investigation and communication time; and a repeat-analysis rate to reagent use, technical time, and TAT. Not every quality indicator should become a financial metric because some are important primarily because of clinical risk. Economic analysis is most useful when it identifies recurrent events for which improvement is likely to release meaningful resources or prevent downstream work.

The practical sequence is to measure the event, identify the recurrent cause, estimate the resources involved, intervene, and measure again. Without intervention and reassessment, quality dashboards remain descriptive rather than managerial tools.

From correction to prevention

Recurrent correction consumes resources while leaving the cause unchanged. For repeated specimen rejection or delayed results, the economically relevant intervention is one that targets a measurable cause and is reassessed after implementation.

Options include collection training, improved identification and transport, validation rules, and workflow redesign. The appropriate investment depends on the frequency, cost, and clinical risk of the local failure.

Automation and autoverification

Automation can reduce manual handling, standardize repetitive processes, and stabilize TAT, but its value should not be judged by throughput alone. Recent discussions of automation in value-based laboratory medicine place greater emphasis on precision, capacity, outcomes, and resource use [22].

Autoverification can release routine results that meet predefined analytical and logical criteria while directing exceptions to professional review. Gao et al. used 1,219,141 historical thyroid-function results to design their system; in a separate post-optimization evaluation, the pass rate rose and the median laboratory turnaround time fell from 122.1 to 88.6 minutes [23]. These observations require local validation and do not make the historical design set an intervention cohort.

Repeat testing provides another example. Işıklar et al. showed that the proportion of repeat results considered unnecessary varies according to the analytical criteria applied, supporting rule-based approaches rather than automatic repetition of every abnormal result [24]. The economic benefit is not simply fewer manual steps; it is a combination of reduced unnecessary work, faster release of routine results, and more professional time for cases that require interpretation.

Artificial intelligence

AI may detect patterns missed by conventional checks, but its clinical and economic value must be demonstrated.

Potential applications include patient-based real-time quality control, specimen-quality assessment, contamination detection, result prioritization, and support for test utilization. In a multicenter study, a machine-learning-based patient-based real-time quality-control model detected simulated analytical biases across 10 measurands better than two comparator models, with validation in three independent hospital datasets [25]. Spies et al. used unsupervised machine learning to identify patterns consistent with intravenous-fluid contamination in basic metabolic panels, showing that clinically relevant preanalytical problems can escape conventional workflows [26].

These studies establish technical potential. Evaluation should also measure false alerts, additional review work, patient-relevant outcomes, and the resources required for implementation and maintenance.

Practical framework for COPLQ

The literature provides extensive information on laboratory errors, quality indicators, utilization, and selected economic consequences, but these elements are often considered separately. For practical purposes, we propose that COPLQ be considered across four components: direct failure cost, workforce and workflow cost, downstream clinical cost, and low-value utilization cost.

Direct failure cost includes materials, reagents, consumables, and analyzer use. Workforce and workflow cost includes laboratory and clinical staff time, specimen collection, transport, investigation, and communication. Downstream clinical cost includes repeat investigations, delayed decisions, additional assessments, and prolonged pathways. Low-value utilization cost includes unnecessary or duplicated testing and the activity it generates.

COPLQ is a practical extension of the CoQ concept for laboratory medicine, not a validated health-economic equation. Its purpose is to make recurrent loss visible. A laboratory does not need to analyze every possible failure. A more realistic approach is to select a small number of frequent or high-risk events, determine their annual frequency, estimate the resources consumed per event, and reassess them after a targeted intervention.

A simple worked example illustrates the approach. Suppose a laboratory records 1,000 hemolyzed specimens in one year. Instead of assigning only the cost of the discarded tube, the laboratory could estimate the average staff time for recollection and accessioning (the extra venipuncture, the second call to a busy ward), additional transport and processing, repeat analytical work when applicable, and the frequency with which the event causes a clinically relevant delay. Multiplying each locally measured component by the annual number of events provides a transparent estimate of recurrent resource consumption. Repeating the calculation after a collection-training or traceability intervention then shows whether the intervention reduced both the quality indicator and the resources consumed. The numerical values are deliberately local; the method, rather than a universal price per error, is the transferable element.

Examples suitable for this approach include hemolyzed specimens from the emergency department, corrected reports, troponin TAT outliers, unnecessary repeat chemistry analyses, or tests ordered before a defined minimum retesting interval. Table 4 summarizes representative studies that illustrate the range of economic perspectives used in this field and the limits of transferring estimates between settings.

**Table 4:** Representative studies informing the economic assessment of poor laboratory quality The studies use different economic perspectives and methods; numerical estimates should not be transferred between settings without local costing and clinical context. CoQ, cost of quality; TRY, Turkish lira; QC, quality control; CAD, Canadian dollar; CPOE, computerized provider order entry

Study	Setting/perspective	Quality or utilization problem	Economic approach/principal finding	Main limitation for transferability
Carlson et al. [3]	Laboratory/pathology process; quality-management perspective	Specimen-process defects and rework	Applied the CoQ framework to make prevention, appraisal, and failure costs visible in laboratory/pathology processes	Framework is highly useful conceptually, but local process structure and costing determine the monetary result.
Green [2]	Preanalytical laboratory processes	Poor blood-specimen quality	Described direct and downstream costs generated by preanalytical error and poor specimen quality	Cost components vary by institution, staffing model, and clinical setting.
Foglia et al. [11]	One Italian university hospital; integrated collection and traceability, 2013–2019	Preanalytical defects and traceability	Reported fewer preanalytical errors and 12.7% cost savings per tube with integrated blood collection and traceability	Single-setting implementation; technology and labor costs are local
Smart et al. [17]	US outpatient claims; payer perspective	2019 claims for four common tests beyond recommended frequencies	Excess direct expenditure exceeded US$350 million for four tests; 7%-51% exceeded recommended frequencies across tests studied	Claims cannot fully capture clinical indication or transfer directly to non-US systems
John et al. [27]	Scoping review	Preanalytical errors	Synthesized consequences for patient care, operational efficiency, and healthcare resource use	Underlying studies were heterogeneous and not designed around a single costing framework.
Devis et al. [28]	Review; combined quality, economic, and environmental perspective	Inappropriate laboratory testing	Linked overuse with economic and environmental consequences in addition to quality-of-care effects	Environmental and economic methods differ markedly across included literature.
Stangalini et al. [29]	One Italian laboratory; 2024 sustainability assessment	Avoidable blood collection and test-ordering practices	Examined economic and environmental burden associated with avoidable activity	Local workflow and sustainability assumptions limit direct transfer of numeric estimates
Eker [12]	One Turkish hospital; 2019 direct-cost perspective	32,783 preanalytical sample errors	TRY 438,284.51 (€68,918.07) estimated direct costs	Single institution and historic prices; downstream clinical costs excluded
Thakur et al. [14]	Canadian laboratory; QC purchasing	Excess control-material use	Annual QC material expenditure declined from CAD 346,395 to CAD 255,267 after process changes.	Local before-and-after comparison; transferability and causal attribution limited
Corchon-Peyrallo et al. [20]	Spanish primary care; 28 centers	Inappropriate and redundant ordering	Median cost/request decreased €5.69 (14%) after laboratory-led CPOE	Before-and-after design and local costing limit causal interpretation

Implementation considerations

A quality intervention that appears attractive in principle may fail if the laboratory lacks the capacity to implement it. Upfront capital expenditure, training time, and interoperability with the laboratory information system all influence whether an intervention produces value. Less tangible factors also matter, including temporary workflow disruption, staff acceptance, and competing operational priorities.

For this reason, implementation should be proportional to the underlying problem. A high-volume recurrent failure may justify automation or major process redesign, whereas an infrequent low-risk event may be better addressed through a focused procedural change. Baseline measurement is essential because the benefit depends on the starting failure rate. Stakeholder involvement is equally important: specimen-collection problems often require nursing or phlebotomy engagement, demand-management initiatives require clinician agreement, and electronic alerts require careful design to avoid unnecessary interruptions.

A phased approach is often more informative than immediate system-wide adoption. Laboratories can begin with one measurable failure, implement a defined intervention in a limited setting, assess its effect on the quality indicator, workload, TAT, and unintended consequences, and then decide whether broader implementation is justified. This is consistent with the central economic principle of the review: prevention should be targeted, measurable, and proportionate to the failure it is intended to prevent.

Discussion

Quality spending is visible in budgets, while repeated work, recollection, investigation, and delay are often distributed across services. Local estimates should identify which failures recur, the resources they consume, and whether targeted interventions reduce both.

Preanalytical quality is an important starting point. Error proportions depend on local detection methods [9], while cost estimates depend on the setting and costing perspective [2,12,17].

Quality activity itself can consume resources. Risk-based controls, validated rules, and focused interventions should be assessed against meaningful clinical and operational outcomes rather than maximized indiscriminately.

Here, the CoQ model and value-based laboratory medicine converge. The former asks where resources are lost; the latter asks whether laboratory activity improves clinical care relative to the resources used. Quality indicators provide a natural bridge because they offer a common language for measuring failure and increasingly international benchmarks for performance [6,16,21]. Their most useful economic role is not to convert every indicator into a financial target, but to identify preventable failures that consume substantial resources or carry significant clinical risk.

Laboratory value must also be judged from the perspective of patients and clinicians. A faster result has limited value if it does not alter management, and a technically accurate result has limited value if the clinician cannot interpret it or does not trust the process that produced it. Conversely, avoiding recollection reduces patient burden, reliable critical-result communication can shorten time to action, and better diagnostic stewardship can reduce unnecessary downstream testing. These consequences reinforce why laboratory quality cannot be assessed solely by analyzer performance or cost per test.

An additional consequence of poor quality is the loss of laboratory capacity. Recollection, repeat centrifugation, repeat analysis, troubleshooting, specimen tracing, communication with clinical units, and unnecessary manual verification occupy staff and instrument time that could otherwise be devoted to new clinical work. This opportunity cost is difficult to identify in conventional accounting because it is absorbed into routine workload, but it is especially relevant in high-volume laboratories with limited personnel or analytical capacity. A recent scoping review of preanalytical quality similarly highlighted operational efficiency and resource use as important consequences of error [27].

The same failures have an environmental dimension. Repeat collection and unnecessary testing consume additional tubes, needles, plastics, reagents, transport, and energy. Contemporary reviews place laboratory overuse within a combined patient-safety, economic, and environmental framework [28], and recent real-world work has examined the burden associated with avoidable blood collection and inappropriate test ordering [29]. Sustainability should not displace patient safety as the primary objective, but preventing repeated work may create clinical, operational, economic, and environmental value at the same time.

Automation and AI require the same evaluation as other interventions: whether they reduce recurrent work or clinically relevant delay at an acceptable implementation and maintenance cost.

The available economic evidence has important limitations. Studies use different costing perspectives, definitions, workflows, currencies, and organizational settings. Some measure only direct laboratory expenditure, whereas others incorporate staffing, claims, or downstream care. Single-center estimates may therefore be highly informative locally but poorly transferable to another health system. Table 4 makes this heterogeneity explicit rather than treating all monetary findings as directly comparable. AI applications also require external validation, monitoring for performance drift and false alerts, transparent governance, and professional oversight.

This narrative review used a focused rather than exhaustive search, restricted its documented update to English-language PubMed records, and did not perform a formal risk-of-bias assessment or a PRISMA screening process. The exact strings and counts of the original search were not available in the retained materials. The revision search is fully documented, but relevant work may still have been missed. The findings support a conceptual and practical synthesis, not a pooled estimate of effect or cost.

The same caution applies to the COPLQ framework proposed here. It is intended as a practical structure for local analysis, not as a validated economic model. Its usefulness should be tested prospectively in different laboratory settings. Nevertheless, laboratories already collect much of the information required to begin: rejection rates, hemolysis, corrected reports, TAT, repeat testing, and critical-result communication. The missing step is often to connect these events with the resources repeatedly spent managing them.

## Conclusions

Poor laboratory quality consumes resources through recollection, repeated analysis, investigation, corrected reports, delays, and inappropriate orders. A practical first step is to identify recurrent, measurable failures, estimate their local consequences, intervene on the cause, and reassess performance.

The COPLQ framework organizes this local assessment but is not yet a validated economic model. Prospective studies should link standardized indicators to resource use and patient outcomes across settings. Technologies and quality measures should be adopted when their measured benefits justify their implementation and maintenance costs.

## Figures and Tables

**Figure 1:**
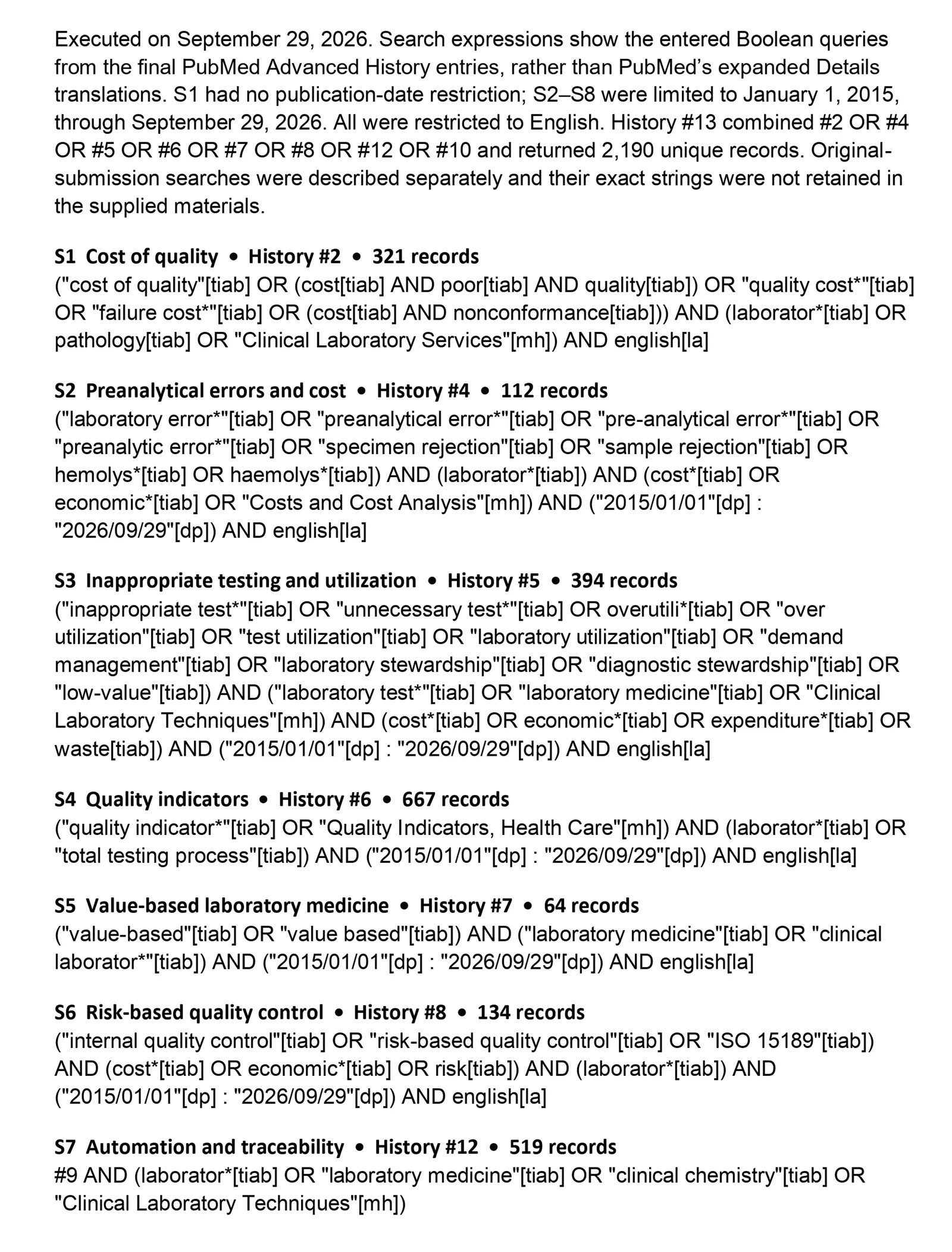
PubMed revision search (Contd.).

**Figure 2:**
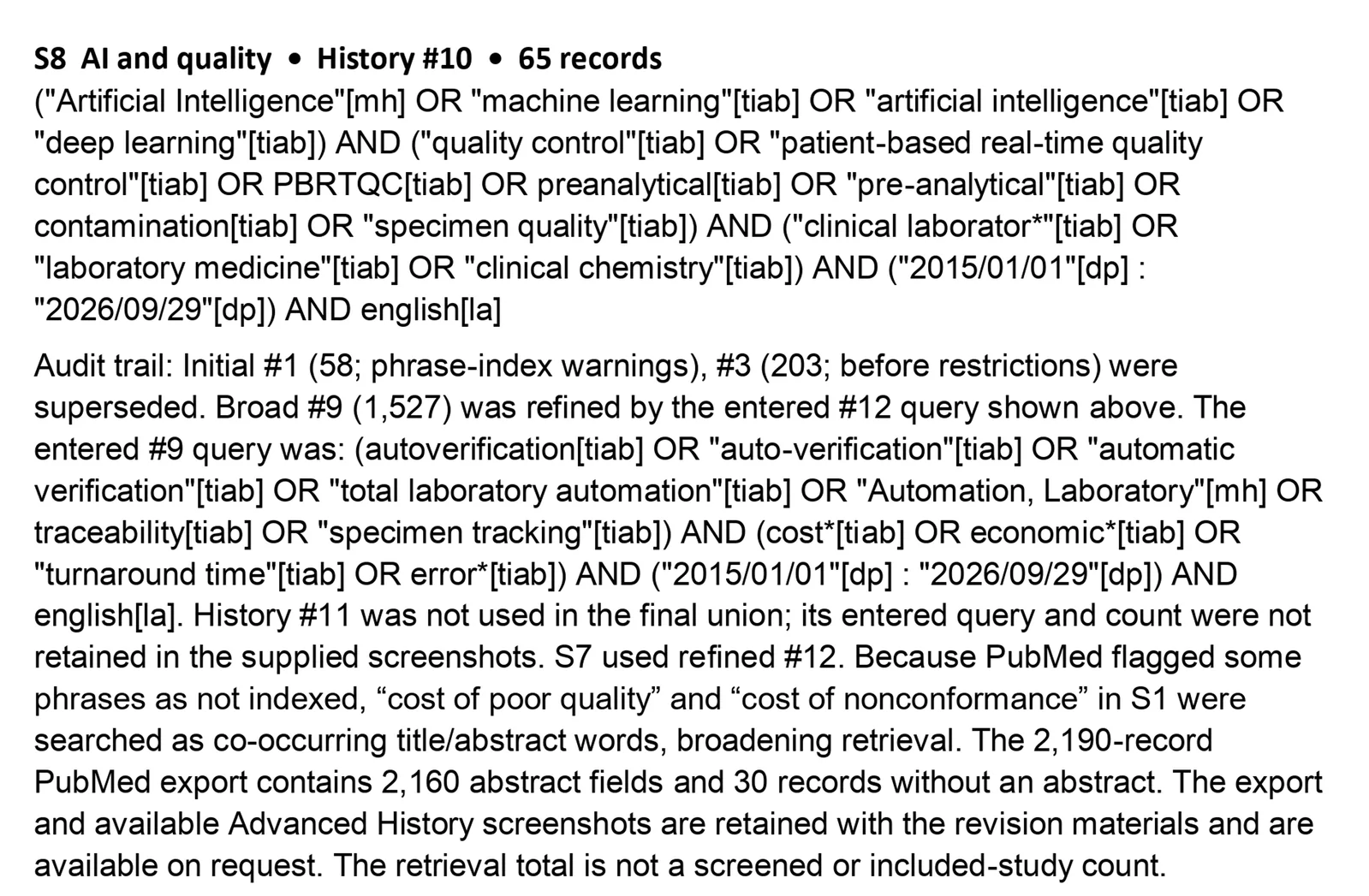
PubMed revision search.

## Appendices

Appendix A

Appendix B 
